# Self-medication and its typology in Chinese elderly population: A cross-sectional study

**DOI:** 10.3389/fpubh.2022.954305

**Published:** 2022-10-19

**Authors:** Shangren Qin, Junjie Zhao, Mengqiu Zhou, Yenuan Cheng, Ye Ding

**Affiliations:** ^1^School of Public Health, Hangzhou Normal University, Hangzhou, Zhejiang, China; ^2^School of Public Health, Hangzhou Medical College, Hangzhou, Zhejiang, China

**Keywords:** self-medication, typology, elderly, China, communication with a doctor, cross-sectional study

## Abstract

**Purpose:**

This paper aims to evaluate the prevalence of self-medication and its associated factors among the Chinese elderly. Also, according to whether the elderly communicate with doctors (no matter before or after self-medication), we aimed to categorize self-medication and explore the associated factors.

**Methods:**

It was a cross-sectional study. Data were derived from the 2018 wave of the China Health and Retirement Longitudinal Study (CHARLS). According to whether communicate with doctors or not, self-medication was reclassified as “self-medicate and NOT communicating with a doctor,” and “self-medicate and communicate with a doctor.” A binary logistic regression was used to identify which elderly were more likely to self-medicate, and a multinomial logistic regression was applied to explore the associated influencing factors of self-medication classifications.

**Results:**

A total of 17,445 individuals aged ≥45 years were enrolled. The prevalence of self-medication was 58.60%. Self-medication was strongly associated with sex, education level, pension, self-reported general health status, chronic illness, satisfaction with local medical services, and three province-level socioeconomic welfare variables. About 19.64% of self-medication populations had communicated with a doctor. Higher education level and younger age were significantly associated with a higher probability of “self-medication and communication with a doctor.”

**Conclusion:**

The prevalence of self-medication among the Chinese elderly is increasing over the year. Health education on appropriate medication use targeting elder adults with low education levels is highly recommended. The typology of self-medication and its factors are new research entry points and could be meaningful for future studies.

## Introduction

Self-medication is defined as “the taking of any drug or medication on one's initiative, or on the advice of another person, for self-diagnosed illness without consulting a doctor” (1). It has some pros and cons. Responsible self-medication can not only empower the public to cure minor ailments themselves, but also save time and money (2). However, many people don't have a clear picture of themselves and self-medicate inappropriately (3). What's more, compared to the medications used with health professionals' prescriptions, self-medication to a certain extent is not confirmed concerning pregnancy, use in children and the elderly, interactions, and so on (3). Although controversial, self-medication is still a worldwide phenomenon, and the prevalence varies in different countries around the world. For example, it was 22% in Spain (4) and 35.9% in Ethiopia (5).

In the twenty-first century, population aging has become a global trend due to the development of medical science. The elderly population is growing faster than other age groups (6). In general, people who live longer tend to suffer from multiple chronic diseases and, consequently pay higher costs for health care treatments (7). Moreover, financial distress and limited mobility can make it difficult for the elderly to seek medical care. Therefore, the elderly sometimes resort to self-medication to ameliorate disease symptoms.

Among the elderly, the prevalence of self-medication was reported from 4 to 87%, and the mean prevalence was 38% (8). For instance, 14.3% of Brazilians aged ≥60 years had reported using drugs without prescription during the 15 days before the interview (9). Likewise, a study conducted in Mexico showed that the reported prevalence of self-medication was 53.5% among the elderly aged >65 years during the last 30 days (10). Moreover, socio-demographic variables associated with elderly self-medication were gender (11, 12), marital status (12), an education level (3, 11), and income (3).

In addition to the studies mentioned above, many papers have studied inappropriate self-medication in the elderly (13). For instance, among the Brazilian elderly who practiced self-medication, 55.5% used inappropriate drugs (14). Inappropriate self-medication includes the following ways: (a) Taking the drugs included on the list of potentially inappropriate medications for the elderly (14); (b) Wrong way of taking drugs (e.g., using excessive dosages) (13); (c) Polypharmacy, Non-essential medication (e.g., irrational use of antimicrobials) (13). Inappropriate self-medication may result in the worsening of the disease, drug interaction, drug toxicity, drug dependence, adverse events, microbial resistance, and so on (15). These inappropriate medication ways are often derived from past medication experiences. So, do the elderly have no communication with doctors before or after self-medication? For instance, before self-medication, the elderly may get information from the doctors that they can self-purchase certain medications; or after inappropriate self-medication, the elderly may have to seek medical service due to the worsening condition. However, previously published self-medication studies focused on its prevalence, reasons, and associated factors. Few studies have explored the doctor-patient communication status before or after self-medication.

Therefore, we conducted a cross-sectional study on self-medication among the Chinese elderly. First, we aimed to evaluate the prevalence of self-medication and its associated factors among the Chinese elderly. Second, we tried to explore the doctor-patient communication status before or after self-medication. Then according to whether the elderly communicate with doctors (not matter before or after self-medication), we aimed to categorize self-medication and explore the associated factors.

## Methods

### Design of the study

This was a cross-sectional study. Its study population was the Chinese elderly aged≥45 years. First, it described the prevalence of self-medication among the Chinese elderly. Then, Andersen's behavioral model-related variables, lifestyle variables, satisfaction variables, and province-level socioeconomic welfare variables were regarded as the independent variables. The status of self-medication was regarded as the dependent variable. After that, a binary logistic regression was used to identify which elderly were more likely to self-medicate. Second, according to whether the elderly communicate with doctors (not matter before or after self-medication), self-medication was reclassified as “didn't self-medicate,” “self-medicate and NOT communicating with a doctor,” and “self-medicate and communicate with a doctor.” Likewise, a multinomial logistic regression was applied to explore the associated influencing factors of the three classifications.

### Data

Data in this study were derived from the 2018 wave of the China Health and Retirement Longitudinal Study (CHARLS). Detailed information on CHARLS can be found on the website http://charls.pku.edu.cn/en/.

Briefly, the CHARLS was a national survey aimed to collect a representative sample of Chinese residents aged ≥45 years to serve the needs of scientific research on the elderly. Its baseline survey was conducted in 2011 and 17,708 individuals aged≥45 years were nationally recruited from 28 provinces, 150 countries/districts, and 450 villages/urban communities. Then it performed wave 2 in 2013, wave 3 in 2015, and wave 4 in 2018. In each wave, a face-to-face computer-assisted personal interview was conducted and a detailed questionnaire was finished on each individual. The questionnaire includes the following modules: demographics, family structure, health status, and functioning, health care and insurance, work, retirement and pension, income and consumption, assets (individual and household), biomarkers, and community-level information. More details on the sampling method, the questionnaire, and the database introduction were available from Zhao et al. (16, 17).

**Figure 1:**
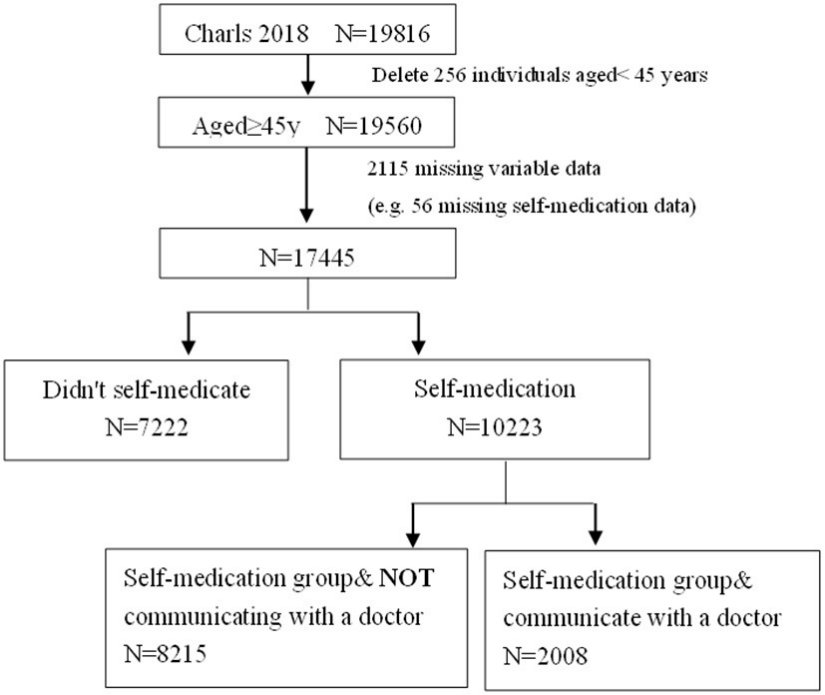
Flow chart of participants through the study.

As shown in Figure 1, there were a total of 19,816 individuals in the 2018 wave of CHARLS, of which 256 were under 45 years. After deleting the individuals aged <45 years, there were 19,560 individuals aged ≥45 years, of which 2,115 were missing some variable data (e.g., 56 were missing self-medication data and 61 were missing income data). Finally, 17,445 individuals were included in our study.

### Measures

#### Self-medication (Dependent variable)

First, self-medication was the dependent variable of the binary logistic regression. It was measured by the question in the CHARLS questionnaire: (1). Did you take any purchased medicine during the past month? (Not including prescription medications, but any medicine delivered by others or stored by oneself is also counted). Those who answered “Yes” were recognized as self-medication cases.

Second, the self-medication classification was the dependent variable of the multinomial logistic regression. In the questionnaire, there was a question about doctor visits: (2) In the last month have you visited a public hospital, private hospital, public health center, clinic, or health worker's or doctor's practice, or been visited by a health worker or doctor for outpatient care (Not including physical examination)? Combined with the above self-medication question, those who answered “No” to question 1 were recognized as “didn't self-medicate.” Those who answered “Yes” to question 1 and “No” to question 2 simultaneously were recognized as “self-medicate and NOT communicating with a doctor.” Finally, those who answered “Yes” to question 1 and “Yes” to question 2 simultaneously were recognized as “self-medicate and communicate with a doctor.” In this study, “communication with a doctor” means “visiting or consulting a doctor for a medical condition.”

#### Andersen's behavioral model-related variables (independent variables)

Andersen's behavioral model has been widely applied in numerous studies for understanding access to and utilization of health services (18, 19). This model suggests that the use of health services in an individual is determined by three key factors: predisposing, enabling, and need factors.

Predisposing factors usually are socio-demographic variables. In this study, the predisposing factors include age, sex, marital status, Hukou (household registration), and education level. The Hukou system in China was created to modernize and manage rural to urban migration. In this system, individuals could only have one place of regular residence. Then according to the registered place of residence, Hukou is divided into two categories: rural Hukou and urban Hukou (20).

Enabling factors refer to resources that can impede or facilitate the utilization of health services. In this study, the enabling factors include individual income, health insurance, and pension. The individual income was calculated as the sum of the individual's wage, retirement pay, and all sources of subsidies or benefits (e.g., elderly family planning subsidies, unemployment compensation, and so on). Then according to the tertiles of income, subjects were divided into three groups: low, medium, and high-income groups.

Need factors represent the need for health care services and are generally related to health status. In this study, the need factors include self-reported general health status and chronic illness.

#### Lifestyle and satisfaction variables (independent variables)

Previous studies have shown that smoking and drinking alcohol might affect one's use of health services (21). These two lifestyles are also reported as influencing factors of self-medication behavior (22). Therefore, smoking and alcohol drinking were included in our study. Each included individual was classified as a current smoker or a non-smoker. Those who had quit smoking were regarded as non-smokers. In the questionnaire, there was a question about alcohol consumption: Did you drink any alcoholic beverages, such as beer, wine, or liquor in the past year? Those who answered “Yes” were identified as current drinkers, otherwise were non-drinkers.

Moreover, several studies had published that dissatisfaction with publicly funded health services was significantly associated with self-medication (23, 24). Therefore, satisfaction with local medical services was added to our independent variables. In the CHARLS questionnaire, individuals were asked about their satisfaction with the quality, cost, and convenience of local medical services. Those who answered “Very satisfied” or “Somewhat satisfied” were regarded as “Satisfied” in our study. Those who answered “Somewhat dissatisfied” or “Very dissatisfied” were regarded as “Dissatisfied.” Those who answered “Neutral” remained in the same group.

#### Province-level socioeconomic welfare variables (independent variables)

Individuals are social actors, residing in social environments that contain different levels of support and resources. Many studies have indicated a key role of social environments in affecting the use of health services (25, 26). Therefore, we planned to add social environment-related variables to this study.

Fortunately, we found an excellent article describing the association between province-level socioeconomic welfare and depression among the Chinese elderly (27). This study also used the 2018 CHARLS data. The research steps of this article are briefly described as follows: (a) Fourteen province-level socioeconomic welfare variables were extracted from the China Civil Affairs Statistical Yearbook for 28 provinces (the same provinces as the CHARLS study). (b) Principal component analysis (PCA) was used to extract three socioeconomic welfare factors constructed from the above 14 province-level variables. (c) These three socioeconomic welfare factors were named economic welfare, social welfare, and medical welfare. (d) A Bayesian mixed-effects logistic model was used to explore the associations between the three socioeconomic welfare factors and depression while controlling for socio-demographic variables. More details of the study can be found in its original text.

In this reference article, the three socioeconomic welfare factor scores for 28 provinces were publicly published and were used as independent variables in our study.

### Statistical analysis

The self-medication and its classification were described, and the Chi-square test was used to examine the statistical difference in self-medication status between socio-demographic variables. The multicollinearity between variables was tested. All values of variance inflation factor (VIF) were <10 which indicated no multicollinearity existed. Then, a binary logistic regression was used to identify which elderly were more likely to self-medicate. Also, a multinomial logistic regression was applied to explore the associated influencing factors of the three classifications. The Odds Ratio (OR) and its 95% confidence interval (95%CI) were calculated.

All statistical tests were 2-sided, and *P* <0.05 were considered statistically significant. Data were statistically analyzed using STATA version 14.0 (STATA Corp, College Station, Texas).

## Results

A total of 17,445 individuals aged ≥45 years were enrolled in this study, including 10,223 (58.60%) who reported self-medication during the past month.

### Self-medication and its associated factors

As shown in Table 1, the results of the Chi-square test indicated that the distribution of self-medication prevalence was different according to various independent variables. For the predisposing factors, a higher proportion of female, older individuals had reported self-medication (both *P* < 0.001). Concerning the enabling factors, those with middle income (*P* < 0.001), having pension (*P* = 0.002) or health insurance (*P* = 0.042) seemed to have a higher prevalence of self-medication. As for the need factors, individuals with poor health status and more chronic diseases tend to self-medicate more frequently. Moreover, individuals without habits of smoking and alcohol drinking reported a higher rate of self-medication (both *P* < 0.001). Also, a higher prevalence was observed among individuals who were dissatisfied with the local medical services (*P* < 0.001).

**Table 1:** Associations between factors and self-medication among elder Chinese, 2018 (*N*, %).

**Factors**	**All sample (*N* = 17,445)**	**Didn't self-medicate**	**Self-medication group**	**OR (95%CI)^a^**
**Sex**				
Male	8,273	3,600 (43.52)	4,673 (56.48)	Reference
Female	9,172	3,622 (39.49)	5,550 (60.51)	1.15 (1.06–1.25)**
**Age group**				
45–59	7,727	3,442 (44.55)	4,285 (55.45)	Reference
60–74	7,793	3,063 (39.30)	4,730 (60.70)	0.99 (0.91–1.07)
75+	1,925	717 (37.25)	1,208 (62.75)	1.08 (0.95–1.22)
**Marital status**				
Married	15,029	6,261 (41.66)	8,768 (58.34)	Reference
Others^b^	2,416	961 (39.78)	1,455 (60.22)	0.93 (0.84–1.02)
**Hukou**				
Rural resident	13,710	5,725 (41.76)	7,985 (58.24)	Reference
Urban resident	3,735	1,497 (40.08)	2,238 (59.92)	1.05 (0.96–1.15)
**Education**				
Illiterate	3,753	1,523 (40.58)	2,230 (59.42)	Reference
Elementary school	7,599	3,083 (40.57)	4,516 (59.43)	1.08 (0.99–1.18)
Middle school	3,887	1,629 (41.91)	2,258 (58.09)	1.13 (1.01–1.26)*
High school	1,844	825 (44.74)	1,019 (55.26)	1.01 (0.88–1.16)
College graduate and above	362	162 (44.75)	200 (55.25)	1.12 (0.87–1.44)
**Income**				
Low	5,937	2,475 (41.69)	3,462 (58.31)	Reference
Middle	5,715	2,152 (37.66)	3,563 (62.34)	1.07 (0.98–1.17)
High	5,793	2,595 (44.80)	3,198 (55.20)	0.99 (0.90–1.08)
**Pension**				
No	1,866	835 (44.75)	1,031 (55.25)	Reference
Yes	15,579	6,387 (41.00)	9,192 (59.00)	1.11 (1.00–1.24)*
**Health insurance**				
No	493	226 (45.84)	267 (54.16)	Reference
Yes	16,952	6,996 (41.27)	9,956 (58.73)	1.13 (0.93–1.37)
**Self-reported general health status**				
Bad	4,585	1,240 (27.04)	3,345 (72.96)	Reference
Fair	8,551	3,475 (40.64)	5,076 (59.36)	0.69 (0.63–0.75)***
Very good/good	4,309	2,507 (58.18)	1,802 (41.82)	0.43 (0.39–0.48)***
**Chronic illness**				
None	3,511	2,204 (62.77)	1,307 (37.23)	Reference
1	4,153	2,015 (48.52)	2,138 (51.48)	1.61 (1.46–1.77)***
≥2	9,781	3,003 (30.70)	6,778 (69.30)	2.82 (2.58–3.08)***
**Smoke**				
No	12,735	5,148 (40.42)	7,587 (59.58)	Reference
Yes	4,710	2,074 (44.03)	2,636 (55.97)	0.99 (0.91–1.08)
**Alcohol drinking**				
No	11,469	4,588 (40.00)	6,881 (60.00)	Reference
Yes	5,976	2,634 (44.08)	3,342 (55.92)	1.03 (0.95–1.11)
**Satisfaction with local medical services**				
Dissatisfied	2,861	941 (32.89)	1,920 (67.11)	Reference
Neutral	7,969	3,319 (41.65)	4,650 (58.35)	0.81 (0.73–0.89)***
Satisfied	6,615	2,962 (44.78)	3,653 (55.22)	0.74 (0.67–0.81)***
**Economic welfare** ^c^	17,445	7,222 (41.40)	10,223 (58.60)	0.78 (0.74–0.83)***
**Medical welfare** ^c^	17,445	7,222 (41.40)	10,223 (58.60)	1.08 (1.03–1.13)**
**Social welfare** ^c^	17,445	7,222 (41.40)	10,223 (58.60)	0.82 (0.78–0.85)***

^a^P for logistic regression. ^*^P < 0.05, ^**^P < 0.01, ^***^P < 0.001.

^b^Include divorced, separated, widowed and single.

^c^Source from the article of Li et al. (27).

Using the binary logistic regression, the associations between factors and self-medication were also analyzed in Table 1. First, the female elderly were more likely to self-medicate than the male elderly (OR = 1.15, 95% confidence interval = 1.06–1.25). Second, compared with the illiterate individuals, those with middle school education levels were markedly associated with higher odds of reporting self-medication [OR (95%CI) = 1.13 (1.01–1.26)]. Third, individuals with a pension or more chronic diseases would report a notably higher rate of self-medication. Fourth, lower odds of self-medication were observed among individuals with better self-reported health status and more satisfaction with local medical services. Fifth, as for the province-level socioeconomic welfare variables, we found increasing economic welfare and social welfare were significantly associated with a lower probability of self-medication [OR (95%CI) = 0.78 (0.74–0.83); OR (95%CI) = 0.82(0.78–0.85)], while medical facilities were associated with a higher probability of self-medication [OR (95%CI) = 1.08 (1.03–1.13)].

### Typology of self-medication and its associated factors

The distribution of self-medication classification was shown in Table 2. According to whether the elderly communicate with doctors during the last month (not matter before or after self-medication), the self-medication was reclassified as “didn't self-medicate,” “self-medicate and NOT communicating with a doctor,” and “self-medicate and communicate with a doctor.” The total number of each group was 7,222 (41.40%), 8215 (47.09), and 2008 (11.51%), respectively. In other words, about 19.64% of self-medication populations had visited a doctor during the same period before the investigation.

**Table 2:** Multinomial logistic regression model for influencing factors of self-medication and doctor communication among elder Chinese, 2018 (reference category is the group who didn't self-medicate).

**Factors**	**Didn't self-medicate**	**Self-medicate & NOT communicating with a doctor**		**Self-medicate & communicate with a doctor**	
	***N* (%)**	***N* (%)**	**OR (95%CI)^**a**^**	***N* (%)**	**OR (95%CI)^**a**^**
**Sex**					
Male	3,600 (43.52)	3,856 (46.61)	Reference	817 (9.87)	Reference
Female	3,622 (39.49)	4,359 (47.53)	1.14 (1.04–1.24)**	1,191 (12.98)	1.21 (1.06–1.39)**
**Age group**					
45–59	3,442 (44.55)	3,368 (43.59)	Reference	917 (11.86)	Reference
60–74	3,063 (39.30)	3,852 (49.43)	1.03 (0.95–1.12)	878 (11.27)	0.78 (0.69–0.89)***
75+	717 (37.25)	995 (51.69)	1.15 (1.01–1.31)*	213 (11.06)	0.77 (0.63–0.94)*
**Marital status**					
Married	6,261 (41.66)	7,051 (46.92)	Reference	1,717 (11.42)	Reference
Others^b^	961 (39.78)	1,164 (48.18)	0.92 (0.83–1.02)	291 (12.04)	0.95 (0.81–1.11)
**Hukou**					
Rural resident	5,725 (41.76)	6,429 (46.89)	Reference	1,556 (11.35)	Reference
Urban resident	1,497 (40.08)	1,786 (47.82)	1.04 (0.94–1.14)	452 (12.10)	1.12 (0.96–1.30)
**Education**					
Illiterate	1,523 (40.58)	1,803 (48.04)	Reference	427 (11.38)	Reference
Elementary school	3,083 (40.57)	3,625 (47.70)	1.06 (0.97–1.16)	891 (11.73)	1.19 (1.03–1.37)*
Middle school	1,629 (41.91)	1,822 (46.87)	1.10 (0.98–1.23)	436 (11.22)	1.29 (1.08–1.54)**
High School	825 (44.74)	810 (43.93)	0.96 (0.83–1.11)	209 (11.33)	1.27 (1.02–1.59)*
College graduate and above	162 (44.75)	155 (42.82)	1.03 (0.79–1.34)	45 (12.43)	1.71 (1.15–2.55)**
**Income**					
Low	2,475 (41.69)	2,695 (45.39)	Reference	767 (12.92)	Reference
Middle	2,152 (37.66)	2,908 (50.88)	1.10 (1.00–1.21)*	655 (11.46)	0.97 (0.84–1.12)
High	2,595 (44.80)	2,612 (45.09)	1.01 (0.92–1.11)	586 (10.11)	0.90 (0.77–1.04)
**Pension**					
No	835 (44.75)	834 (44.69)	Reference	197 (10.56)	Reference
Yes	6,387 (41.00)	7,381 (47.38)	1.11 (0.99–1.24)	1,811 (11.62)	1.13 (0.95–1.35)
**Health insurance**
No	226 (45.84)	220 (44.62)	Reference	47 (9.53)	Reference
Yes	6,996 (41.27)	7,995 (47.16)	1.11 (0.91–1.36)	1,961 (11.57)	1.21 (0.86–1.70)
**Self-reported general health status**					
Bad	1,240 (27.04)	2,423 (52.85)	Reference	922 (20.11)	Reference
Fair	3,475 (40.64)	4,173 (48.80)	0.77 (0.70–0.84)***	903 (10.56)	0.47 (0.42–0.53)***
Very good/good	2,507 (58.18)	1,619 (37.57)	0.52 (0.47–0.58)***	183 (4.25)	0.18 (0.15–0.22)***
**Chronic illness**					
No chronic disease	2,204 (62.77)	1,170 (33.33)	Reference	137 (3.90)	Reference
One chronic disease	2,015 (48.52)	1,828 (44.02)	1.56 (1.41–1.72)***	310 (7.46)	2.07 (1.67–2.56)***
Two chronic diseases and more	3,003 (30.70)	5,217 (53.34)	2.54 (2.32–2.78)***	1,561 (15.96)	5.07 (4.18–6.16)***
**Smoke**					
No	5,148 (40.42)	5,984 (46.99)	Reference	1,603 (12.59)	Reference
Yes	2,074 (44.03)	2,231 (47.37)	1.03 (0.95–1.13)	405 (8.60)	0.79 (0.68–0.91)**
**Alcohol drinking**					
No	4,588 (40.00)	5,427 (47.32)	Reference	1,454 (12.68)	Reference
Yes	2,634 (44.08)	2,788 (46.65)	1.05 (0.97–1.13)	554 (9.27)	0.93 (0.82–1.06)
**Satisfaction with local medical services**					
Dissatisfied	941 (32.89)	1,488 (52.01)	Reference	432 (15.10)	Reference
Neutral	3,319 (41.65)	3,756 (47.13)	0.83 (0.75–0.91)***	894 (11.22)	0.73 (0.64–0.85)***
Satisfied	2,962 (44.78)	2,971 (44.91)	0.75 (0.68–0.83)***	682 (10.31)	0.68 (0.59–0.79)***
**Economic welfare** ^c^	7,222 (41.40)	8,215 (47.09)	0.78 (0.73–0.83)***	2,008 (11.51)	0.78 (0.71–0.87)***
**Medical welfare** ^c^	7,222 (41.40)	8,215 (47.09)	1.08 (1.03–1.14)**	2,008 (11.51)	1.07 (0.99–1.15)
**Social welfare** ^c^	7,222 (41.40)	8,215 (47.09)	0.81 (0.78–0.84)***	2,008 (11.51)	0.85 (0.79–0.91)***

^a^P for logistic regression.^*^P < 0.05,

^**^P < 0.01,

^***^P < 0.001.

^b^Include divorced, separated, widowed and single.

^c^Source from the article of Li et al. (27).

We also conducted a multivariate multinomial logistic regression to explore the associated factors. In the regression model, those who didn't self-medicate were regarded as the reference category. Similar results of associated factors were found in both the “self-medicate and NOT communicating with a doctor” group and the “self-medicate and communicate with a doctor” group. For example, female individuals and those with more chronic diseases reported a higher rate of self-medication. Likewise, in both groups, we found those with better self-reported health status and more satisfaction with local medical services were less likely to self-medicate. Economic welfare and social welfare were also significantly associated with a lower probability of self-medication.

However, there were still differences in associated factors between these two self-medication groups. For instance, the regression results revealed that younger age and higher educational level were associated with a higher probability of “self-medication and communication with a doctor.” But in the “self-medication and NOT communicating with a doctor” group, a higher odds ratio was found among individuals aged >75 years, and no significant associations were observed with education levels. In addition, middle income and medical welfare were significantly associated with a higher probability of “self-medication and NOT communicating with a doctor.” While in the “self-medication and communication with a doctor” group, we couldn't observe similar results.

## Discussion

In this study, we found the prevalence of self-medication was 58.60% among the Chinese elderly in 2018. However, using the data derived from CHARLS 2011 and 2013, a published study has reported that the prevalence of self-medication is 32.69% for over-the-counter medicines (OTCs) and 15.02% for prescription-only medicines (POMs) among the Chinese elderly (22). The definition of self-medication in the above-published study is the same as ours. But it divides the self-medicating drugs into OTCs and POMs, and the total prevalence of all drugs' self-medication is not reported. Although the prevalence in our study couldn't be directly compared with that in the published study, we still find the prevalence of 58.60% in 2018 is greater than the sum of prevalence in 2011 and 2013. Therefore, the prevalence of self-medication is increasing among Chinese elderly aged ≥45 years. Under the same definition of self-medication, the prevalence (58.60%) among Chinese elderly is lower than that of Brazilian elderly (68%) (28). However, the prevalence of self-medication between China and other countries is still hard compared due to the different definitions of self-medication.

In our study, we found that self-medication was strongly associated with several factors such as sex, education level, pension, self-reported general health status, chronic illness, satisfaction with local medical services, and three province-level socioeconomic welfare variables. First, sex and education level are predisposing factors of Andersen's behavioral model. Consistent with previous research (11, 12, 29), the results showed females and those with higher education levels resorted to self-medication more frequently than others. Second, income, pension, and health insurance are enabling factors of Andersen's behavioral model. Several studies have described that people with higher income (3, 30) or without health insurance (11, 31) are more likely to self-medicate. However, no significant associations between income, health insurance, and self-medication were indicated in our study. Third, self-reported general health status and chronic illness are need factors of Andersen's behavioral model. In our study, people with worse self-reported health status or more chronic diseases were reported to be more likely to self-medicate, and this finding was consistent with other studies (32). There might be two explanations: One is that people with poor health status may have limited mobility, making it difficult to see a doctor. They have to self-medicate to ameliorate disease symptoms. The other is that the people with more diseases may have more experience in treatment, and try to self-medicate for old weaknesses. They see no need for visiting a doctor. Fourth, people who are more satisfied with local medical services are more likely to use medical treatment rather than self-medication. Fifth, negative associations were detected between economic welfare, social welfare, and self-medication in our study. Generally speaking, the better the economic welfare and social welfare in an area, the more convenient the transportation and the more developed the informatization. Therefore, it is convenient to seek medical treatment rather than self-medicating. While better medical welfare means more hospitals are available, there are also more pharmacies and more ways to buy medicines. In addition, high-quality medical resources will attract more seriously ill patients from other areas. For common minor problems, local people may be more willing to go to the pharmacy to buy medicine, rather than crowding the hospital to see a doctor.

Most of the influencing factors of the two self-medication classifications were basically the same. Two obvious differences were: (a) education was significantly associated with “self-medication and communication with a doctor,” not with “self-medication and NOT communicating with a doctor.” (b) Older people were more likely to “self-medicate and NOT communicate with a doctor,” while younger people were more likely to “self-medicate and communicate with a doctor.” In other words, individuals with younger ages and higher education levels were more frequently communicating with a doctor no matter before or after self-medication. There are two possible explanations for these differences: (a) Individuals with younger age and higher education levels generally have better communication skills than those with older or lower education levels. They are better able to explain themselves to their doctors if they had problems before and after self-medication. (b) Individuals with younger age and higher education levels are more capable of deciding about their own health-related needs (4, 33). Once they have problems derived from self-medication, they can decide more quickly whether to seek a doctor's help than those who are older or less educated.

The strength of our study is the large national sample used, which ensures the representativeness of the sample and sufficient statistical power. We have included many reasonable factors to find out which are the best predictors for self-medication. Most important of all, we have explored the relationship between self-medication and doctor communication, and also reclassified self-medication according to whether the elderly communicate with doctors (not matter before or after self-medication). This is the first study on self-medication and doctor communication among the Chinese elderly. Nevertheless, the study has several limitations. First, it is a cross-sectional study and the observed associations may not be causal. Second, due to the limited information provided by the questionnaire, we cannot know why and when the patients communicated with their doctors. In other words, we cannot know whether self-medication occurred before or after doctor communication. Third, information about drugs and their sources for self-medication, and reasons for self-medication are not collected in our study.

## Conclusion

In summary, the prevalence of self-medication was 58.60% among the Chinese elderly and was increasing over the years. Self-medication was strongly associated with several factors such as sex, education level, pension, self-reported general health status, chronic illness, satisfaction with local medical services, and three province-level socioeconomic welfare variables. Moreover, about 19.64% of self-medication populations had communicated with a doctor. Higher education level and younger age were significantly associated with a higher probability of “self-medication and communication with a doctor.”

These findings can provide theoretical bases for self-medication studies. Previously published self-medication studies focused on its prevalence, reasons, and associated factors. Few studies have explored the doctor-patient communication status before or after self-medication. In this study, self-medication was classified according to whether communicate with a doctor or not. Education and age were also found associated with “self-medication and communication with a doctor.” These discoveries are novel, and we believe the typology of self-medication and its factors are new research entry points and could be meaningful for future self-medication studies, as well as doctor-patient communication studies.

Moreover, our findings have important health policy implications for China. Health education on appropriate medication use targeting elder adults with low education levels is highly recommended. Local economic welfare, social welfare, and people's satisfaction with local medical services should be improved, which will help reduce the prevalence of self-medication. In pharmacies, stricter implementation of prescription-only regulations should be enforced to reduce the source of self-medication. The prevalence of “self-medication and communication with a doctor” was not low in our study, suggesting that self-medication-related problems (e.g., adverse events, inappropriate drug use) should be paid attention to.

## Data availability statement

Publicly available datasets were analyzed in this study. This data can be found here: The datasets 2018 Charls for this study in a public database, and can be found on the website http://charls.pku.edu.cn/en/.

## Ethics statement

Ethical approval for human participation in the CHARLS project was granted from the Institutional Review Board at Peking University. The participants provided their written informed consent to participate in the CHARLS survey.

## Author contributions

SQ conceived the idea and design of this study. JZ, MZ, and YC further improved the quality of manuscript writing. YD dealt with data analysis and wrote the manuscript. All authors contributed to the article and approved the submitted version.

## Funding

This study was funded by the Soft Science Research Program of Zhejiang Province (Grant No: 2022C35064), the Medical and Health Technology Plan Project of Zhejiang Province (Grant No: 2022RC126), and the General Project of the Department of Education of Zhejiang Province (Grant No: Y202249243). The financial sponsor played no role in the design of the study and collection, analysis, and interpretation of data, and the writing of the manuscript.

## Conflict of interest

The authors declare that the research was conducted in the absence of any commercial or financial relationships that could be construed as a potential conflict of interest.

## Publisher's note

All claims expressed in this article are solely those of the authors and do not necessarily represent those of their affiliated organizations, or those of the publisher, the editors and the reviewers. Any product that may be evaluated in this article, or claim that may be made by its manufacturer, is not guaranteed or endorsed by the publisher.
